# Two-Plasmid Packaging System for Recombinant Adeno-Associated Virus

**DOI:** 10.1089/biores.2020.0031

**Published:** 2020-10-16

**Authors:** Qiushi Tang, Allison M. Keeler, Songbo Zhang, Qin Su, Zhuoyao Lyu, Yangfan Cheng, Guangping Gao, Terence R. Flotte

**Affiliations:** ^1^ Department of Pediatrics, University of Massachusetts Medical School, Worcester, Massachusetts, USA.; ^2^ Horae Gene Therapy Center, University of Massachusetts Medical School, Worcester, Massachusetts, USA.; ^3^ Vector Core, University of Massachusetts Medical School, Worcester, Massachusetts, USA.; ^4^ Department of Microbiology and Physiology Systems, University of Massachusetts Medical School, Worcester, Massachusetts, USA.

**Keywords:** AAV, viral vectors, plasmids, gene expression, gene therapy, gene transfer

## Abstract

A number of packaging systems are available for production of recombinant adeno-associated virus vectors (rAAVs). Among these, the use of a two-plasmid cotransfection system, in which Rep and Cap genes and Ad helper genes are on the same plasmid, has not been frequently employed for good manufacturing practices (GMP) production, even though it presents some practical advantages over the common three-plasmid (triple) transfection method. To confirm and expand the utility of the two-plasmid system, we generated GMP-compatible versions of this system and used those package reporter genes in multiple capsid variants in direct comparison with triple transfection. Vector yields, purity, and empty-to-full ratios were comparable between double and triple transfection methods for all capsid variants tested. We performed an *in vivo* side-by-side comparison of double and triple transfection vectors following both intravenous injection and intramuscular injection in mice. Expression and transduction were evaluated in muscle and liver 4 weeks after injection. Additional studies of bioactivity were conducted *in vivo* using packaged vectors carrying a variety of cargos, including the therapeutic transgene, microRNA, and single- or double-stranded vector. Results showed that cargos packaged using double transfection were equivalently bioactive to those packaged using a triple transfection system. In conclusion, these data suggest the utility of midrange (1E12-1E16) GMP-compatible packaging of adeno-associated virus (AAV) vectors for several AAV capsids.

## Introduction

The production of recombinant adeno-associated virus (AAV) vectors in the relative absence of wild-type AAV was first accomplished by Samulski, Chang, and Shenk using cotransfection of adenovirus 5-infected HeLa cells with an AAV2 inverted terminal repeat (ITR)-flanked vector cassette and AAV2 ITR-deleted construct to provide *rep* and *cap* functions *in trans.*^1^ Initially, it was assumed that supplying greater levels of expression of both the Rep78/68 and Rep52/40 proteins would increase packaging. However, it was subsequently shown that highly regulated *rep* gene expression (particularly moderating the expression of Rep78/68) would increase the yield of rAAVs packaged under such conditions.^2^ A further substantial improvement in the efficiency of the upstream process was achieved by substituting transfection of three of the adenoviral helper genes (*E2a, E4*, and *VA* RNA) into cells that constitutively express adenoviral E1a and E1b genes, most commonly HEK-293 cells.^3^ This process of triple transfection of the ITR-flanked vector plasmid, Rep/cap plasmid, and adenoviral gene helper plasmid has become an established and highly flexible manufacturing method. Grimm et al. further improved this approach by combining the Rep/cap and adenovirus helper gene capsids into a single helper, pDG.^4,5^ However, this two-plasmid system has not been used as frequently for clinical manufacturing as the common triple transfection system or the baculovirus system.^6–13^

The recent emergence of numerous instances of clinical efficacy being proven in rAAV-mediated gene therapy trials for single-gene disorders has precipitated a greatly increased demand for current good manufacturing practices (cGMP) reagents that will enable this platform to be disseminated more broadly to the estimated 10,000 distinct monogenic disorders of humans.^14–24^

We sought to develop a cost-sensitive, streamlined good manufacturing practices (GMP)-compatible set of reagents that would enable rapid cross-translation of rAAV platform technologies from one monogenic disease to other monogenic diseases affecting the same organ or cell type.^25^ These constructs were derived using a strategy similar to that of Grimm et al.,^4^ with minor backbone modifications to improve GMP compatibility and allow the insertion of multiple, different, serotype capsid genes that would be of interest in clinical trials. In such an approach, clinical success with a particular rAAV capsid variant, route, and dose combination in one genetic disease affecting one cell type would provide a strong rationale for its use in the treatment of other genetic diseases affecting the same cell type, so long as the same capsid/route/dose combination was used.

Obstacles to this approach might include the availability of reagents needed to manufacture rAAVs in a relatively versatile and low-cost manner. To address these obstacles, we tested a set of GMP-compatible rAAV packaging plasmids that combine a number of the features of previously reported technology. In this study, we report that the use of a two-plasmid system instead of a three-plasmid system simplifies the transfection step and decreases by one-third the cost of manufacturing the GMP-compatible plasmid, thus resulting in significant cost savings in the cGMP packaging of rAAVs.^26^

## Results

**FIG. 1.**
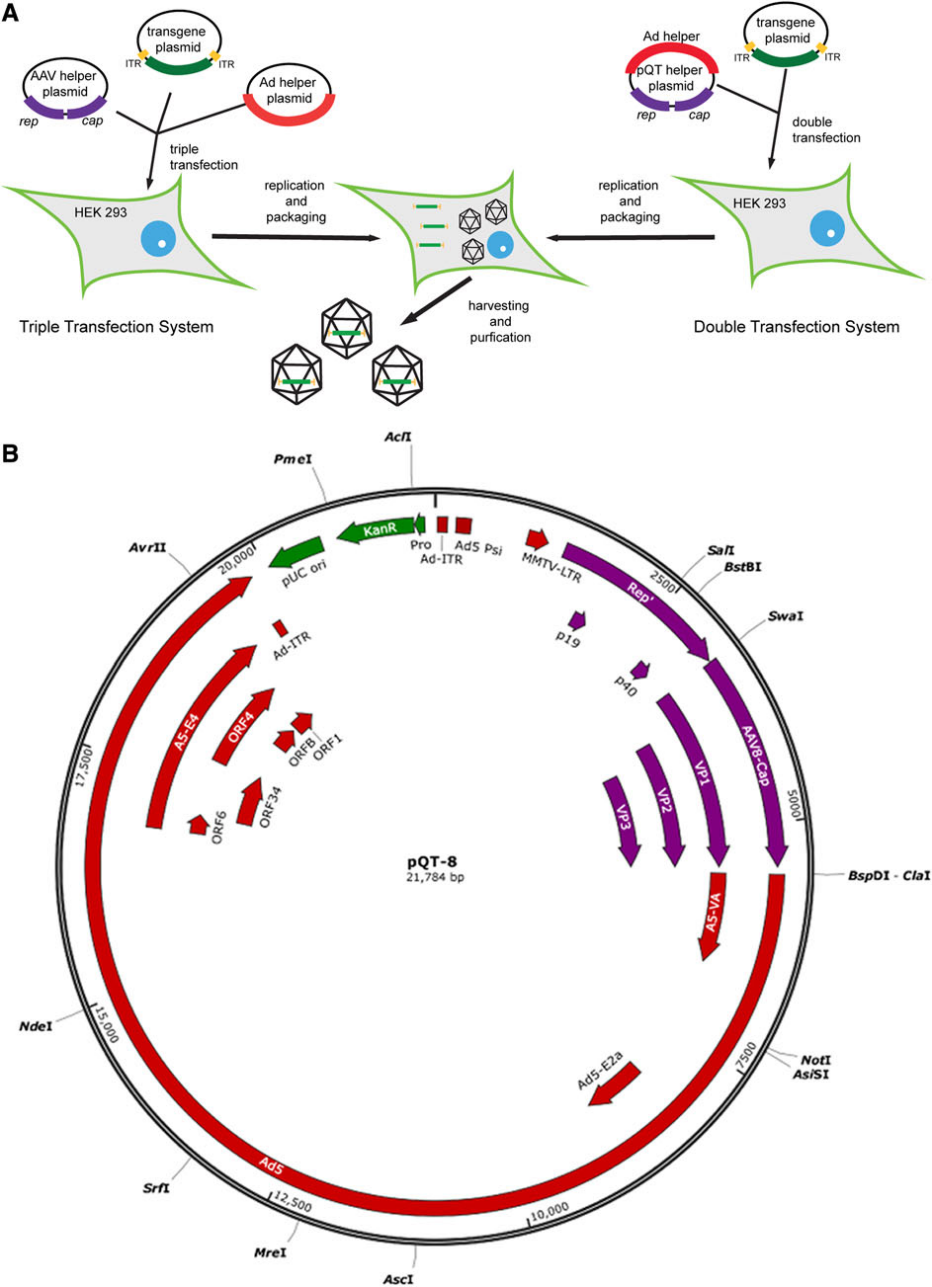
pQT plasmid packaging system. Red—adenoviral (Ad) genes, purple—AAV genes. **(A)** Schematic comparing the standard triple-plasmid transfection system (right) with the double-plasmid transfection using the pQT helper plasmid. ITRs designated in yellow, and transgene of interest in green. **(B)** Plasmid map of the pQT plasmid. Bacterial genes designated in green. AAV, adeno-associated virus; ITRs, inverted terminal repeats.

To create a two-plasmid system for AAV packaging, the Ad5 helper genes were cloned into a plasmid containing ITR-less AAV Rep and Cap genes, creating the pQT-packaged plasmid that contains both AAV Rep and Cap and the Ad helper function (Fig. 1). Importantly, kanamycin resistance was used for bacterial selection to make the pQT plasmid appropriate for clinical use. To package rAAVs using this system, the pQT plasmid is transfected with the transgene plasmid flanked by AAV ITRs (Fig. 1A). To create multiple AAV capsid variants from this two-plasmid system, separate pQT helper plasmids must be created for each capsid and are therefore referred throughout the article with the nomenclature of pQT-8 representing the two-plasmid helper system for packaging the AAV capsid variant AAV8 (Fig. 1B).

**FIG. 2.**
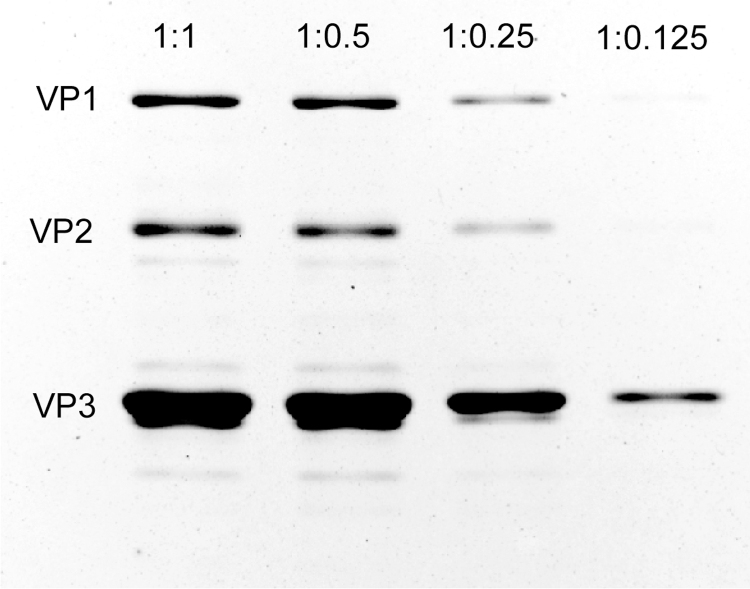
Silver-stained protein gels of viral capsid proteins from vector preparations with two-capsid systems. Silver stain of SDS-PAGE gel of purified pQT8 virus packed at different ratios of the transgene plasmid: pQT packaging plasmid. SDS-PAGE, sodium dodecyl sulfate–polyacrylamide gel electrophoresis.

To determine the optimal ratio of the two plasmids for transfection, four different molar ratios (1:1, 1:0.5, 1:0.25, and 1:0.125, transgene:helper) of pQT-8 were transfected into HEK cells and viruses were purified as previously described. Silver staining of vector particles indicates that ratios of 1:1 and 1:0.05 (transgene plasmid: pQT-8 plasmid) produce the greatest number of viral particles as well as minimal contaminating bands (Fig. 2). Digital droplet polymerase chain reaction (PCR) was used to determine genome copies per prep and confirmed that ratios of 1:1 and 1:05 produced the best yield. The results are summarized in Table 1, indicating the vector particle and genome copy per mL and total yield per prep at different molar ratios. There are instances where the two titer methods differ slightly in their values in either direction (physical vs. genome copy), but these differences appear to be within the error of the measurement technique.

**Table 1.** Optimization of Different Molar Ratios for pQT Packaging

Ratio	1:1	1: 0.5	1: 0.25	1: 0.125
VP/mL	5.0 × 10^11^	1.0 × 10^12^	1.0 × 10^12^	1.0 × 10^11^
GC/mL	1.6 × 10^12^	1.9 × 10^12^	7.2 × 10^11^	9.7 × 10^10^
Volume, mL	2.4	2.4	2.4	2.0
Total yield (GC)	3.8 × 10^12^	4.6 × 10^12^	1.7 × 10^12^	1.9 × 10^11^

Ratio of transgene plasmid: pQT packaging plasmid.

Genome copies (GC) were detected by digital droplet PCR and viral particles (VP) were detected by silver stain.

Next, to compare the pQT system with the well-established triple transfection system, different AAV capsid variants were packaged side by side using either the pQT helper or triple transfection helper plasmids. pQT plasmids were packaged at a molar ratio of 1:1 and triple transfection packaging used a 1:1:1 weight ratio of plasmid, in which the weight of the transgene plasmid was matched between the pQT method and triple transfection system.

**FIG. 3.**
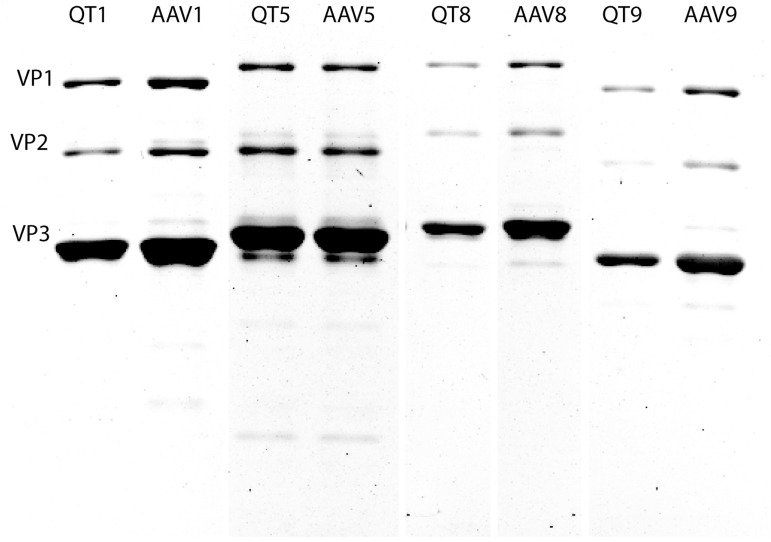
Silver-stained protein gel of viral capsid proteins from multiple capsid variants. After purification of AAV particles by either the traditional triple transfection system or pQT packaging, 5 μL of virus was run on SDS-PAGE gel and stained with silver stain. VP1, VP2, and VP3 capsid proteins are designated on the gel. Quantification of gel staining was preformed and recorded as viral particles per mL in Table 1.

To assess the applicability of the pQT method across different plasmid variants, AAV1, AAV5, AAV8, and AAV9 capsids were tested comparing the two packaging systems. Viral particle production was analyzed by silver staining for the different capsid variants packaged by either pQT or triple transfection (Fig. 3). Results showed that expression levels of viral capsids using pQT or triple transfection were similar, although some of the triple transfection AAVs were more concentrated than the pQT-packaged AAVs, resulting in higher viral particles (VP)/mL. This difference did not, however, correlate with a higher total yield (Table 2). Visualization of AAV capsids by transmission electron microscopy after purification demonstrated that the capsid structure and ratio of empty to full capsids were comparable across the two transfection systems and different capsids (Supplementary Fig. S1). Genome copies were again compared, and total yield was calculated and is reported in Table 2. Across all capsid variants, pQT and triple transfection titers were similar and within normal deviations of viral production.

**Table 2.** Viral Titer Comparison

Capsid variants	AAV1		AAV5		AAV8		AAV9	
Transfection	Triple	pQT	Triple	pQT	Triple	pQT	Triple	pQT
VP/mL	2.4 × 10^13^	1.3 × 10^13^	1.8 × 10^13^	2.3 × 10^13^	1.0 × 10^13^	8.0 × 10^12^	9.0 × 10^12^	5.5 × 10^12^
GC/mL	4.2 × 10^12^	5.2 × 10^12^	6.7 × 10^12^	7.6 × 10^12^	4.6 × 10^12^	3.0 × 10^12^	6.5 × 10^12^	2.5 × 10^12^
Total volume (mL)	2.9	1.9	3.0	3.2	2.0	3.0	2.0	3.0
Total yield (GC)	1.2 × 10^13^	9.9 × 10^12^	2.0 × 10^13^	2.4 × 10^13^	9.2 × 10^12^	9x 10^12^	1.3 × 10^13^	7.5 × 10^12^

Genome copies (GC) were detected by digital droplet PCR and viral particles (VP) were detected by silver stain.

**FIG. 4.**
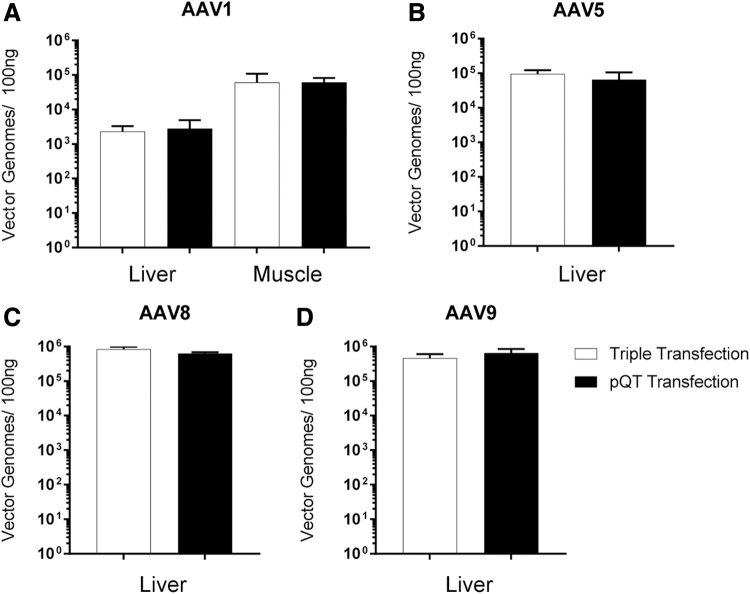
Vector genome quantification after *in vivo* delivery of different capsid variants. **(A)** 1E10 vg were intramuscularly injected into four animals per group. Tissue was collected from the muscle injection site and liver and vector genomes were analyzed by qPCR targeting polyA tail. **(B–D)** 1E11 vg were intravenously injected into four animals per group. Liver tissue was collected and vector genomes were analyzed by qPCR targeting polyA tail. qPCR, quantitative polymerase chain reaction.

**FIG. 5.**
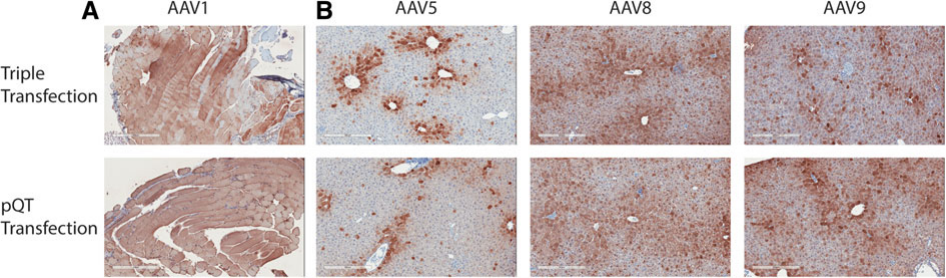
Immunohistochemical detection of GFP expression in the liver and muscle after *in vivo* AAV administration of multiple capsid variants. DAB staining of GFP expression (brown) within the liver **(A)** and muscle **(B)** after AAV administration through intramuscular injection (AAV1) or intravenous injection (AAV5, AAV8, and AAV9). All scale bars represent 300 μm.

Next, we wanted to test how the pQT-packaged viruses behaved *in vivo*. C57bl6 mice were injected with either pQT-produced or triple transfection-produced AAVs through the tail vein for AAV5, AAV8, and AAV9 (1 × 10^12^ vg) or through intramuscular injection for AAV1 (1 × 10^11^ vg). Three weeks postinjection, animals were sacrificed, and tissues were collected for quantification of vector genomes and immunohistochemistry. Measurements for AAV1 vector genomes in liver and muscle showed no significant differences between the two methods (Fig. 4A). For AAV5, 8, and 9, tissues were collected from the liver and again no significant differences were observed in the vector genomes between the two methods of viral production (Fig. 4B–D). Expression of GFP within the tissue was visualized by immunohistochemistry staining within the muscle (Fig. 5A) or liver (Fig. 5B). Widespread expression was observed within the muscle after treatment using AAV1 vectors packed by both systems (Fig. 5A). Although different expression patterns were observed in the liver among capsid variants (with mostly perivascular cuffing observed for AAV5), the expression did not vary as a function of the transfection method (Fig. 5B) and no detectable differences were observed between transduction and expression of AAV capsids *in vivo*.

**FIG. 6.**
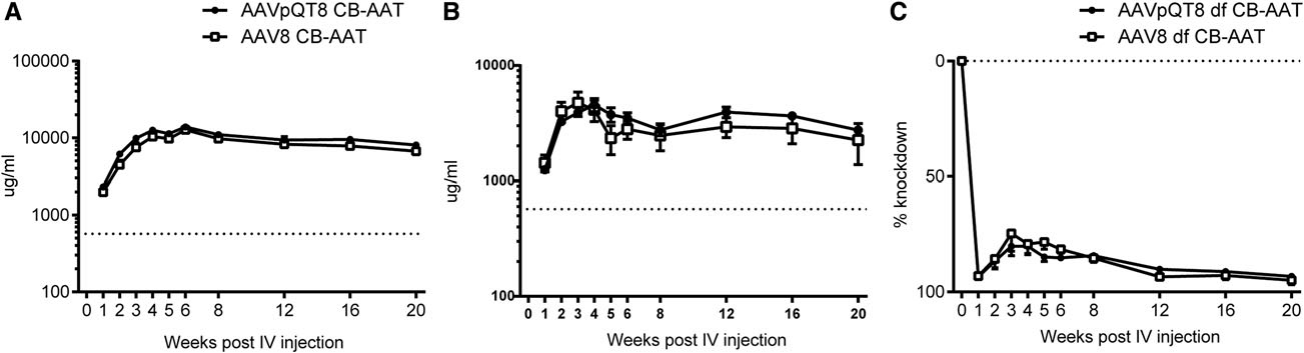
Total serum AAT ELISA after delivery of pQT8 or AAV8-expressing alpha-1-antitrypsin. Rag^−/−^ mice were injected with 5E11 vg intravenously through the tail vein **(A)** with the vector encoding the AAT gene that was packed with either the pQT (circles) or standard triple transfection (squares) method. Dual-function vector contains the alpha-1-antitrypsin gene with the c-myc tag and microRNA targeting mutant PiZ allele. PiZ transgenic mice were injected with 5E11 vg through the tail vein with the dual-function vector packed by either the pQT (circles) or standard triple transfection (squares) method. **(B)** Expression levels of AAT by the surrogate c-myc marker. **(C)** Decrease in expression of the PiZ protein level in the PiZ mouse model. Dotted line **(A, B)** designates 11 μM, which is the level used by protein replacement therapies to determine licensure for human use. **(C)** Baseline expression of the PiZ protein. ELISA, enzyme-linked immunosorbent assay.

Finally, to test the *in vivo* functionality of transgenes packaged by the pQT system, AAV8 vectors were packaged by pQT or the triple transfection system to produce the human alpha-1 antitrypsin (AAT) transgene. Rag^−/−^ mice were injected intravenously with vectors packaged either by the pQT or triple transfection method and bled biweekly to measure AAT levels in the serum. No significant differences were observed in serum AAT levels of vectors packaged by the two methods and injected intravenously (Fig. 6A).

To further show that the pQT vector system could efficiently package transgenes, even those with complex structures such as microRNAs, vectors were packaged using both methods with a transgene that contained AAT with a c-myc tag along with a microRNA targeting the Z mutation of human AAT. Vectors prepared using the two methods were then administered to PiZ transgenic animals, which allowed us to test for both transgene-mediated augmentation of AAT by c-myc using the enzyme-linked immunosorbent assay (ELISA) and microRNA-mediated knockdown of PiZ. Results showed that treatment with pQT-generated vectors led to both increased ATT expression (Fig. 6B) and knockdown of PiZ (Fig. 6C), with no significant difference being found between the two packaging methods. No differences were observed in biological activity of the virus regardless of the production method.

## Discussion

In this study, we demonstrate that a two-vector system can be used to produce AAV that behaves similarly to triple transfection-produced virus, both in its ease of production and its biological activity *in vivo*. Dirk Grimm first published the use of a two-vector system, pDG, combining packaging and helper functions,^4,5^ which when scaled for clinical production, allows for significant cost savings in production of two plasmids versus three plasmids. We have further optimized this system for GMP-compatible, medium-scale clinical production by introducing kanamycin as the bacterial antibiotic resistance gene on the pQT plasmid.

The Grimm Laboratory pDG constructs demonstrated the feasibility of a two-plasmid approach some two decades ago, and yet this approach has still not become the predominant approach to transient transfection-based rAAV manufacturing. It is not clear why this is the case, but the current report shows how this approach can be built upon to yield a lower cost method that allows for great flexibility in the choice of AAV capsid serotype in production of AAV vectors. This improvement should hasten the speed toward clinical development of any number of rAAV constructs. Our experience with contract manufacturing organizations indicates a standard cost based on the number of plasmids. While these cost savings could potentially be offset if yields of plasmids with the pQT series were lower than with alternative plasmids, this has not been our experience (Supplementary Table S1).

It has been the aspiration of many rare disease researchers to reduce rAAV gene therapy for autosomal recessive disorders to a single GMP compatible platform regardless of serotype, dosage, and route of administration.^25^ For this concept to truly become a reality, it will require a generic manufacturing platform such as the one described here. We currently have generated the following pQT plasmids to represent a number of clinically relevant capsid variants: AAV1 (pQT-1), AAV3b (pQT-3b), AAV5 (pQT-5), AAV7 (pQT-7), AAV8 (pQT-8), AAV9 (pQT-9), AAV-RH8 (pQT-RH8), AAV-RH10 (pQT-RH10), and AAV-PHP.*B* (pQT-PHP.B).

The work to optimize plasmid transfection-based manufacturing of rAAVs, while hampered by limitations of transient transfection, nonetheless provides us with an important platform for disseminating this mode of therapy to many individuals suffering from single-gene disorders.

There is no question that the availability of a readily translatable platform for rAAV gene therapy is important to the future of gene therapy. The University of Massachusetts Medical School (UMMS) platform is readily available for licensure to any academic medical center with potential interest in pursuing rAAV gene therapy. The plasmids created here could help in creation of AAV vectors for the treatment of single-gene disorders and beyond.

## Materials and Methods

### Design of plasmids

The pQT vector is designed by cloning AAV Rep and Cap genes from the AAV packaging plasmid and Ad helper genes onto a backbone plasmid containing the kanamycin-resistant gene. The sequences chosen were similar to those described by Grimm et al.^4^ The plasmids were constructed using GenScript cloning. The entire fragment from *Avr*II to *Sal*I was used to substitute the kanamycin resistance gene in the place of the ampicillin resistance gene. In addition, a *Cla*I site was engineered 3′ to the AAV cap sequence to enable the introduction of various new capsid sequences, including those for AAV1, AAV3B, AAV5, AAV8, AAV9, AAVrh8, AAVrh10, and PHP.B. See Figure 1 for schematic representation. These constructs included sequences of the adenovirus ITRs, but these were not tested specifically for rescue as this was not a primary research question for these studies.

### Virus production

The constructs were packaged into AAVs by transient HEK 293 cell (triple or double) transfection and purified by CsCl sedimentation as previously described by the viral vector core at the University of Massachusetts Medical School,^27^ a method based on transfection of HEK293 cells grown in roller bottles, with 1 × 10^8^ cells per bottle and 10 bottles per prep. Triple transfection was performed at a 1:1:1 weight ratio with 1.5 mg each of the transgene plasmid, packaging plasmid, and adeno helper plasmid (unless otherwise specified). pQT packaging was performed at different molar ratios (as stated in text) with 1.5 mg of the transgene plasmid matched to the triple transfection system.

### Titration of vector preparations

The vectors were titered for genome titer by droplet digital PCR (QX200 ddPCR system; Bio-Rad) using the EGFP or SV40 prime and probe. Probes were used with the Bio-Rad protocol for droplet digital PCR and TaqMan assay master mix. All amplifications were performed in 20-μL reaction volumes run for 40 cycles. The following primer–probe combinations were used: eGFP-F: AGCAAAGACCCCAACGAGAA, eGFP-R: GGCGGCGGTCACGAA, and eGFP probe: 6FAM-CGCGATCACATGGTCCTGCTGG-TAMRA; and SV-F: AGCAATAGCATCACAAATTTCACAA, SV-R: CCAGACATGATAAGATACATTGATGAGTT, and SV probe: 6FAM-AGCATTTTTTTCACTGCATTCTAGTTGTGGTTTGTC-TAMRA. AAV testing samples and a reference standard AAV of known titer were treated with DNase I first to eliminate residual vector plasmid DNA and then with proteinase K to destroy DNase I and release DNase I-resistant packaged vector genomes, followed by heat inactivation of proteinase K. Serially diluted AAV samples and the reference standard were each subjected to droplet PCR in a 20-μL reaction volume. The PCR conditions are 10 min at 95°C, followed by 40 cycles of 30 sec at 94°C and 1 min at 60°C, ending with 10 min at 98°C. Semiquantitative capsid titer and purity (vector particles) were assessed by 4–12% sodium dodecyl sulfate (SDS)–acrylamide gel electrophoresis and silver staining (Invitrogen). A precalibrated AAV vector with a known vector genome titer and *a* > 95% full-to-empty ratio was used as a reference standard, serially diluted, and loaded side by side with testing articles of AAV samples onto SDS-polyacrylamide gel electrophoresis (SDS-PAGE) for analysis. After silver staining of the gel, purity of each AAV testing article was visually examined for the presence of AAV VP1, VP2, and VP3 protein bands only at a ratio of 1:1:10, respectively, but absence of other protein bands. The capsid titer of each AAV testing article was semiquantitatively determined on a gel scanner. Briefly, the band size and intensity of VP3 protein of the serially diluted reference standard AAV were measured to establish a standard curve, then the VP3 band size and intensity of each testing article were measured and its capsid titer semiquantitatively assessed by using the reference standard curve.

### Animals

All experimental procedures were approved by the Institutional Animal Care and Use committee at the University of Massachusetts Medical School. Rag mice were obtained from Jackson Laboratory B6.129S7-Rag1<TM1MOM>/J. The PiZ transgenic mice were used in this study, as has been previously described.^28^ Intramuscular injections were administered into the right hindlimb at 50-μL volume, as previously described.^29^ Intravenous injections were administered into the tail vein at a volume of 200 μL per mouse.

### Vector genomes

Vector genomes were determined as previously described.^30^ DNA was extracted from the liver and muscle using the DNeasy Blood and Tissue Kit (Qiagen). The following RGB poly A primer/probe was used: probe 56-FAM/ATGAAGCCCCTTGAGCATCTGACTTCT/36-TAMSp, F: ATTCCAACACACTATTGCAATG, R: GCCAAAAATTATGGGGACAT. The standard curve for quantitative polymerase chain reaction was generated from the same pCB-EGPFP that was packaged and normalized to genome copies per 100 ng genomic DNA.

### GFP immunohistochemistry

GFP immunohistochemistry was carried out by the Molecular Pathology Core at University of Florida. Briefly, 4-mm serial sections were deparaffinized and treated with citra (BioGenex, Fremont, CA) at 98°C for 30 min. Background Sniper (Biocare Medical, Walnut Creek, CA) was used to reduce unspecific background staining. Sections were incubated with rabbit anti-GFP (1:2000; Abcam, Cambridge, MA) for 60 min. The stain was visualized using MACH 2 Gt × rabbit HRP polymer (Biocare Medical), DAB chromogen (Biocare Medical), and CAT hematoxylin counterstain (Biocare Medical).

### Enzyme-linked immunosorbent assays

For all ELISAs (human AAT, human PiZ, and c-myc), high binding 96-well plates (Immulon4; Dynatech Laboratories) were coated with the relevant capture antibody in 100 μL of Voller's buffer overnight at 4°C. All subsequent incubations were done at room temperature for 1 h and plates were washed with phosphate buffered saline containing 0.05% Tween 20. Detection antibody was used at 1:5000 goat anti-hAAT-horseradish peroxidase (HRP, Cat#A80-122P; Bethyl Laboratories, Inc.).

Human AAT ELISA: As previously described,^31^ blocking was done with 1% milk; 1:000 capture antibody goat anti-human AAT (Cat#A80-122A; Bethyl Laboratories, Inc.) was used. Human plasma-purified AAT was used to create the standard curve (Cat#16-16-011609; Athens Research and Technology).

Human PiZ-AAT ELISA: Blocking was done with 3% bovine serum albumin (BSA); 1:000 dilution custom capture antibody was produced in our laboratory (UMMS-PiZ-mAb). In-house standard was used to create the standard curve.

c-myc ELISA: Blocking was done with 3% BSA; 1:1000 c-myc capture antibody (Cat#GTX30518; GeneTex, Inc.) was used. In-house standard was used to create the standard curve.

## Supplementary Material

Supplemental data

Supplemental data

## Abbreviations Used

AAT: alpha-1 antitrypsin
AAV: adeno-associated virus
BSA: bovine serum albumin
cGMP: current good manufacturing practices
GC: genome copies
GMP: good manufacturing practices
HRP: horseradish peroxidase
ITRs: inverted terminal repeats
qPCR: quantitative polymerase chain reaction
rAAV: recombinant adeno-associated virus vector
SDS: sodium dodecyl sulfate
SDS-PAGE: sodium dodecyl sulfate–polyacrylamide gel electrophoresis
UMMS: University of Massachusetts Medical School
VP: viral particles
