# Supplementary material for: Two-Plasmid Packaging System for Recombinant Adeno-Associated Virus
Source: Biores Open Access. 2020 Oct 16;9(1):219–28. doi: 10.1089/biores.2020.0031 (PMC7590824; doi:10.1089/biores.2020.0031)
Supplement: Supplemental data [file Tang_Rev_FigLegSupplem.docx]

**Supplemental Figure and Methods:**

**

**

**Supplemental Figure 1: Electron Microscopy of different AAV capsid variants**

AAV1, AAV5, AAV8 and AAV9 viral preps created by either triple transfection (top) or double pQT transfection (bottom) were imaged by electron microscopy for structural assessment. Micrographs were taken at 87,000 X, and 200nm scaled bars are depicted.

**Electron microscopy Negative Staining:** Electron microscopy negative staining for AAV morphology stained as previously described ^1^. In brief, all spreads are done on freshly prepared Carbon stabilized Formvar Support films on 200 mesh copper grids. The AAV particles were adsorbed onto a carbon-coated Formvar support films for 30 seconds. Excess liquid is removed with filter paper and the sample was immediately negatively stained by running 6 drops of 1% uranyl acetate over the grid to contrast the spread virus particles. Excess stain is removed, and the sample was air dried in a controlled humidity chamber. The samples were then examined using a FEI Tecnai 12 G2 Spirit transmission electron microscope equipped with a Gatan, Erlanshen CCD camera. Micrographs were taken at 87 KX to record the fine structure of the virus particles.

**Supplemental Reference:**

1. Hendricks G, Sena-Esteves M, Gao G. Analysis of Recombinant Adeno-Associated Virus (rAAV) Sample Morphology Using Negative Staining and High-Resolution Electron Microscopy. Cold Spring Harb Protoc. 2020;2020(8):pdb prot095661.
