# Supplementary material for: Two-Plasmid Packaging System for Recombinant Adeno-Associated Virus
Source: Biores Open Access. 2020 Oct 16;9(1):219–28. doi: 10.1089/biores.2020.0031 (PMC7590824; doi:10.1089/biores.2020.0031)
Supplement: Supplemental data [file Tang_Rev_SupplementalTable1.docx]

**Supplemental Table**

| **Plasmid** | **Maxi-Prep Volume** | **Maxi-Prep Yield, mg per liter** |
| --- | --- | --- |
| pQT8 | 3 liter | 5.3 |
| pQT9 | 3 liter | 5.0 |
| pQT1 | 3 liter | 6.3 |
| pQT3B | 3 liter | 8.9 |
| pQT5 | 3 liter | 4.9 |
| pQT8 | 3 liter | 13.1 |
| pQT-Rh8 | 3 liter | 5.3 |
| pQT-Rh10 | 3 liter | 8.3 |
| pQT-PHP.B | 3 liter | 5.8 |
| Conventional Rep2Cap8 (trial 1) | 6 liter | 14.0 |
| Conventional Rep2Cap8 (trial 2) | 6 liter | 5.8 |
| Conventional Rep2Cap8 (trial 3) | 4 liter | 12.0 |
| Conventional Rep2Cap6 | 6 liter | 15.0 |

**Supplemental Table S1:** Yields of various pQT series plasmids in comparison with conventional (non-Ad gene containing) packaging plasmids grown in the same time period and the same laboratory.
